# Social representations of nurses. Differences between incoming and outgoing Nursing students

**DOI:** 10.17533/udea.iee.v38n1e05

**Published:** 2020-02-26

**Authors:** Franco Bastias, Itatí Giménez, Pablo Fabaro, José Ariza, María José Caño-Nappa

**Affiliations:** 1 Psychologist, Psychology Ph.D. student. Professor, Catholic University of Cuyo, San Juan, Argentina. Email: francobastias@uccuyo.edu.ar Universidad Católica de Cuyo Catholic University of Cuyo San Juan Argentina francobastias@uccuyo.edu.ar; 2 Nurse, National University of San Juan, San Juan, Argentina. Email: itatigimenez@gmail.com Universidad Nacional de San Juan National University of San Juan San Juan Argentina itatigimenez@gmail.com; 3 Nurse. National University of San Juan, San Juan, Argentina. Email: pablofabaro@gmail.com Universidad Nacional de San Juan National University of San Juan San Juan Argentina pablofabaro@gmail.com; 4 Nurse, Masters student. Professor, National University of San Juan, San Juan, Argentina. Email: lic.arizajose@hotmail.com.ar Universidad Nacional de San Juan National University of San Juan San Juan Argentina lic.arizajose@hotmail.com.ar; 5 Nurse, Masters in Nursing. Professor, National University of San Juan and Catholic University of Cuyo, San Juan, Argentina. Email: enfermeria.investiga.sj@gmail.com Universidad Nacional de San Juan National University of San Juan San Juan Argentina enfermeria.investiga.sj@gmail.com

**Keywords:** social perception, students, nursing, teaching., percepção social, enfermeiras e enfermeiros, estudantes de enfermagem, ensino.

## Abstract

**Objective.:**

This article explored and compared social representations of nurses held by incoming and outgoing Nursing students in the Technical Nursing Program in San Juan, Argentina.

**Methods.:**

Our research was descriptive and utilized the prototypicality method of analysis for social representations, from a structural approach. The sample was made up of 194 students (104 incoming and 90 outgoing), to whom we applied the word association technique for the term “nurse”.

**Results.:**

Differences were found in the representations that incoming and outgoing students had. i) For incoming students: we observe a wide and general concept of a nurse, expressed in non-specific terms such as “health” in the central core, while for outgoing students the term “care” emerged; ii) We infer distancing from the hegemonic medical model on the part of outgoing students, as well as an emphasis on the relational, as terms such as “vocation”, “humanization”, “love” and “empathy” are evoked, while the term “illness” decreases; iii) We understand that outgoing students highlight their autonomy with respect to doctors and nursing as a profession with the term “professional” with no mention of “assistance”, “help” and “assistant”, terms which did appear with incoming students; iv) Outgoing students convey a sense of a nurse’s diverse roles that go beyond the hospital setting, as instead of mentioning “hospital” and “injection” like incoming students, they mention “prevention” and “research”.

**Conclusion.:**

The comparison of representative structures held by incoming and outgoing students suggests a transformation of self-image through a process of academic education.

## Resumo

**Objetivo.:**

Este estudo investigou e comparou as representações sociais sobre o enfermeiro de estudantes que ingressaram ou saíram do Curso Superior de Enfermagem, em San Juan, Argentina.

**Métodos.:**

A pesquisa foi descritiva e utilizou o método de análise prototípica de representações sociais, a partir da abordagem estrutural. A amostra constituiu 194 alunos (104 participantes e 90 de graduação), aos quais a técnica de associação de palavras foi aplicada contra o termo indutor “enfermeiro”.

**Resultados.:**

Diferenças nas representações do enfermeiro são encontradas entre os estudantes que entram e saem: i) Nos participantes, observa-se uma concepção ampla e geral do enfermeiro, expressa em termos inespecíficos como “saúde” no núcleo central, enquanto nos que estão se formando "cuidado" emerge; ii) Infere-se um afastamento do modelo médico hegemônico por parte dos graduados e uma ênfase no relacional, evocando "vocação", "humanização", "amor" e "empatia" e diminuindo a aparência de "doença" ; iii) entende-se que os estudantes de graduação destacam sua autonomia perante o médico e a enfermagem como profissão, evocando "profissional" e sem mencionar "assistência", "ajuda" e "auxiliar", assim como os participantes; iv) os estudantes que se formaram expressam a diversidade de papéis do enfermeiro além do ambiente hospitalar, em vez de evocar “hospital” e “injeção” como participantes, mencionam “prevenção” e “pesquisa”.

**Conclusão.:**

A comparação das estruturas representativas dos estudantes que entram e saem sugere uma transformação da auto-imagem através do processo de formação acadêmica.

## Introduction

Over the last few decades in Argentina, the nursing world has undergone different social, political and educational processes, all linked to an alarming deficit and scarcity of nursing professionals in health centers. New norms at the regulatory level have given the nursing discipline greater opportunities both in academia and in the professional realm. In academia, efforts have been directed at the professionalization of nursing, as only 11% of nursing personnel in Argentina have university credentials (approximately 5 years of academic instruction), 41% have technical credentials (approximately 3 years of instruction) and 48% are medical assistants (approximately 1 year of instruction).(1) In the workplace, especially in hospitals, work has been done to bring to light and act on the working conditions of nursing staff, their precarious job situation and the lack of professional and social recognition.(2,3) In this sense, the job of a nurse is conditioned by the way in which the profession is seen, giving it a certain role with specific functions, assigning it a status within the interdisciplinary health team and legitimizing and justifying a certain salary and certain physical spaces.(4) For example, in the city of Buenos Aires, strong demands were recently made for the recognition of nurses as health professionals rather than technical-administrative personnel, a category that limits their salary and workplace possibilities.(5) In this context of change, which is also occurring at the international level, the nursing discipline finds itself in a stage of transition, undergoing processes of transformation of the representation of nurses themselves, both inside and outside their professional group.

The theory of social representations(6,7) is presented as an adequate theoretical framework for understanding the thoughts of individuals in society and, in this case, for understanding who a nurse is and what makes someone a nurse for a certain social group. This study seeks to carry out critical-reflexive research on the social representations of nurses that nursing degree students have. We have chosen this population based on the fact that the construction of professional identity begins during the academic period and then wields a strong influence during the entire professional career.(8) Students entering the degree program, far from being mere receptors of a university education, are active co-constructors of their education and their academic process. From the beginning, they attend university not only with their previous academic knowledge, but also with motivations and expectations regarding their professional education, and with representations of the role and function of the professional they will become. In general, this type of knowledge is not evaluated and explored during the program, where education seems to be limited to “academic” knowledge. Nevertheless, beliefs, expectations and social representations of the profession and its work have a relevant role for future professional performance, as they may limit or broaden one’s opportunities.(9)

The objective of this research is to compare the social representations of nurses that incoming nursing students hold with those that outgoing students of the Nursing Technical Program, a 3-year program, hold. We expect that the symbolic and subjective world of the student will be transformed during their time at university through the negotiations that the learning process generates and requires. Nonetheless, this transformation will not only take place in connection with formal education, but also in connection with the different actors that participate in the education process -especially professors who serve as models-, with professional practice and with the exchange that occurs with society itself while the student acquires a new professional identity.(10-13)

## Methodology

Study type and participants. We carried out a descriptive and transversal study, with a non-probabilistic sample. One hundred ninety-four university students from the province of San Juan, Argentina participated in the study. Of the total, 90 were finishing the last semester of the 3-year program to obtain their Technical Degree in Nursing, while 104 were participating in the entrance course to access the program. The participants were invited to take part in the study voluntarily and anonymously. The signing of an informed consent form was required. 

Data collection. Data collection was carried out in San Juan, Argentina in February (for incoming students) and October (for outgoing students) of the year 2017. Using the University’s facilities, participants were gathered in classrooms and given written surveys to complete. These surveys contained questions regarding sociodemographic variables and university program information and instructions corresponding to the word association technique:(7) “Please write the first five words that come to mind when you think of a nurse”.

Data analysis. A prototypicality analysis was carried out to define the structure of the social representation of “nurse”. This lexicographic analysis allows us to obtain an organization of content of the social representations by considering how frequent items appear in the sample and their range of association. This range refers to the order in which words were mentioned. We infer that the earlier the mention or appearance, the greater its importance. If five words are to be mentioned, a range of one or near one means greater importance and a range of five or near five means lesser importance. According to this analysis, those words that appear with greater frequency and importance will make up the *central core* of the representation; those with greater frequency but lesser importance will make up the *first periphery*; the terms with lesser frequency but greater importance will conform the *contrast zone*; and lastly, those with lesser frequency and importance will form the *second periphery*. IRaMuTeQ 0.7 Alpha 2 software was used to complete this analysis.

## Results

194 individuals participated in the study, 104 incoming students and 90 outgoing students from the Nursing Technical Program. General characteristics of the incoming group include: 71% female with an average age of 22.7±5.5 (minimum = 17 and maximum = 40). General characteristics of the outgoing group include: 73% female with an average age of 23.7±4.5 (minimum = 20 and maximum = 42).

Incoming students to the Nursing Technical Program produced 519 words, of which 125 are different words; that is, an average of 1.20 different words per person. For their part, outgoing students of the program produced 450 words, of which 107 are different; that is, an average of 1.18 different words per person. The words obtained were put through a lemmatization process in which they were reduced according to gender (masculine-feminine), number (singular-plural) and semantic context of belonging (see Table 1). In all cases, we opted to use the term that was most frequent to represent less frequent terms of the same semantic context.

**Table 1:** Reduced forms and semantic context

Word	Semantic Context
assist+	to assist
	assistance
help+	help
	to help
care+	care
	to care
heal+	to heal
	healing
	healer
educat+	educator
	to educate
humani+	humanization
	humanized
	humanity
	humanitarian
injec+	injection
	injections
	injectable

For the incoming group, the prototypicality analysis (See Table 2) indicated that the central core of the social representation of “nurse” is made up of the words health, assistance, care and responsibility. Whereas for the outgoing group, the core is made up of the terms care, professional, love, humanization, vocation and health (See Table 3). Although the term health appears in both groups, for incoming students it has a greater percentage of appearance (59% of the subsample total) and greater importance (range = 2.4) as compared to outgoing students (20%, range = 2.8). The opposite happens with the notion of care, which has greater mention and greater importance for outgoing students (*f* = 57; range = 1.6) than for incoming students (*f* = 38; range = 2.8). Other differences in the central core are the presence of the concepts of responsibility and assistance for the incoming group and of the concepts professional, love, humanization and vocation for the outgoing group.

**Table 2:** Social representation of “nurse” for incoming students to the Nursing program

	Range < 2.83	Range < 2.83	Range < 2.83	Range ≥ 2.83	Range ≥ 2.83	Range ≥ 2.83
*f* ≥ 14.85	*Central core*	*f*	*Range*	*First periphery*	*f*	*Range*
*f* ≥ 14.85	health	57	2.4	hospital	24	3.5
*f* ≥ 14.85	assist+	43	2.5			
*f* ≥ 14.85	care+	38	2.8			
*f* ≥ 14.85	responsibility	31	2.8			
14.85 < *f*	*Contrast Zone*	*f*	*Range*	*Second periphery*	*f*	*Range*
14.85 < *f*	heal+	14	2.6	illness	13	3.1
14.85 < *f*	professional	10	2.1	solidarity	12	3.1
14.85 < *f*	love	10	2.6	wellbeing	12	3.8
14.85 < *f*	life	9	2.6	help+	12	3.0
14.85 < *f*	vocation	8	2.2	work	12	3.5
14.85 < *f*	service	8	2.0	dedication	12	3.2
14.85 < *f*	aid	7	1.7	effort	11	3.4
				injec+	9	3.1
				comradeship	8	4.1
				respect	6	3.8
				patience	6	3.2
				hand washing	5	3.2
				culture	5	3.6
				attention	5	3.0

*Note.* Terms with a frequency of less than five are not considered in the table.

For incoming students, the first periphery is made up only of the word hospital, while for outgoing students it includes the terms commitment, empathy, assist and knowledge. For its part, the contrast zone for the incoming group is made up of the terms heal, professional, love, life, vocation, service and assistant; and for the outgoing group, heal and patient.

**Table 3:** Social representation of “nurse” for outgoing students

	Range < 2.83	Range < 2.83	Range < 2.83	Range ≥ 2.83	Range ≥ 2.83	Range ≥ 2.83
*f* ≥ 14.57	*Central core*	*f*	*Range*	*First periphery*	*f*	*Range*
*f* ≥ 14.57	care+	57	1.6	committment	24	3.1
*f* ≥ 14.57	professional	24	2.6	empathy	19	3.1
*f* ≥ 14.57	love	22	2.0	assist+	19	3.0
*f* ≥ 14.57	humani+	20	2.6	knowledge	16	3.3
*f* ≥ 14.57	vocation	19	2.2			
*f* ≥ 14.57	health	18	2.8			
14.57 < *f*	*Contrast zone*	*f*	*Range*	*Second periphery*	*f*	*Range*
14.57 < *f*	heal+	6	2.7	comradeship	14	4.2
14.57 < *f*	patient	6	2.7	solidarity	13	3.7
14.57 < *f*				educa+	10	3.2
14.57 < *f*				respect	7	4.7
14.57 < *f*				research	7	3.7
14.57 < *f*				work	6	3.8
14.57 < *f*				technique	6	3.3
14.57 < *f*				dedication	6	4.5
14.57 < *f*				prevention	6	3.3
14.57 < *f*				illness	5	4.0
14.57 < *f*				predisposition	5*	4.0

*Note.* Terms with a frequency of less than five are not considered in the table.

Lastly, for incoming students, the second periphery includes the terms illness, solidarity, wellbeing, help, work, dedication, effort, injection, comradeship, respect, patience, hand washing, culture and attention. For outgoing students, it includes comradeship, solidarity, education, respect, research, work, technique, dedication, prevention, illness and predisposition.

## Discussion

At present, nursing as a profession and as a discipline is undergoing an important transformation that not only implies new positioning in academia and in the professional realm, but also in relation to its identity. Nevertheless, what has been achieved is often not enough to modify the perception that society, health workers and nurses themselves traditionally have of the profession, a profession seen many times as being a subsidiary of medicine, without autonomy and centered on the hospital. This search for the specification of the role, which implies establishing the tasks, practices and functions of a nurse with the greatest regularity possible, is especially important during the period of university education.(11) In this sense, studying the processes of education and transformation of what it means to be a nurse during academic formation may help us to understand future professional practice, the group’s direction, its public image and its struggle for better positioning within the health system. Our research seeks to make a contribution in this sense, comparing the social representations of nurses that incoming and outgoing students of the Technical Nursing Program have. 

When comparing the social representations of both groups, we point out that incoming nursing students seem to have a broad and general concept of what it means to be a nurse. This can be deduced by looking at the predominance of the term “health” in this group, mentioned by more than half of the sample (59%). This word could also be associated with a large number of health professions. On the other hand, outgoing students mention the word “care” much more than their counterparts, giving it greater importance (according to its range) and greater frequency (mentioned by 63% of the subsample), while relegating the term “health” to only 20% of the group. In any case, health and care are the only terms observed in the central core of both representations. In effect, the healthcare of human beings is the epistemic object of nursing. This notion, located in the central core, is strongly linked to the identity and collective memory of a social group and is resistant to the immediate context of starting or finishing a university program. The relevance of the notion of care reported in this study coincides with that of previous studies. For example, Ten Hoeve et al. found care to be the factor that most influenced the development of the self-concept and professional identity of nurses.(14) Likewise, using the word association technique, Roland-Lévy and Mounguenguila found that the term “care” was the term most mentioned by professional nurses.(15)

Outgoing students characterize and relate nursing to vocation, humanization and love, locating these terms in the core of their representation. Likely humanization, whose semantic field includes the term humanized, characterizes care and other nursing tasks; instead of focusing on illness as does the hegemonic medical model, here it is centered on the patient as an individual and on therapeutic connections. The associations love and empathy, located in the first periphery, are coherent with this idea and the term “illness” appears less for outgoing students than for incoming students. The importance of the relational dimension of their profession for the group as a whole has been highlighted in the work of Roland-Lévy and Mounguenguila, where the words welcome, listen, empathy and relationship were four of the five words most mentioned by French and Gabonese nurses when describing their profession.(15) On the other hand, as opposed to incoming students, outgoing students emphasize the professional nature of their future role, mentioning the word “professional” (term with the second greatest frequency in this subgroup). In that regard, we consider that mentioning the term “professional” could be unnecessary as it is seen as an obvious characteristic of a university program. Nevertheless, this word does make sense if it is thought of as confirming and reaffirming the present processes of the professionalization of nursing.

Now it is interesting to note how three elements (professional, love and vocation) found in the core of the outgoing students’ representation are found in the contrast zone of the incoming students’ representation. In accordance with the central core theory,(7,16) this could indicate the presence of a controversial representation within the incoming group that is fighting for its place against the hegemony. In this case, a representation associated with the nurse’s role itself would be opposing a representation of the nurse from the hegemonic medical model. At the same time, the contrast zone could reflect the perspective of a subgroup within the incoming group who use terms of great importance to refer to themselves but, due to a small number of members, do not achieve high frequency. Therefore, the results suggest that towards the end of the university program this minority becomes a majority and/or this representation, previously controversial, becomes hegemonic.

It is important to highlight the presence of certain elements in the incoming group’s structure that are absent in the outgoing group’s structure. If we compare both cores, we see that the ideas of responsibility and assistance are found with incoming students but not for outgoing students. Perhaps concern for responsibility, if it does occur, is less for advanced students who have already achieved a certain level of self-confidence. However, the absence of the term “assistance” in the central core of outgoing students is noteworthy and could indicate progressive distancing from the idea of nurses being “doctors’ assistants”. In this sense, at least three terms can be understood: the concept of “help” (which could be both “helping the patient” and “helping the doctor”), frequent with the incoming group and absent from the outgoing group, the idea of “assisting” and “service”, both terms of great importance according to their range for incoming students but absent for outgoing students.

As has been mentioned, some studies point to the lack of knowledge that society in general has about nursing, with many believing that nursing is strictly administering injections, unaware of tasks such as prevention, care or healthcare promotion.(14,17,18) In this sense, we note that the term “injection” does appear nine times for incoming students but is completely absent from the outgoing group. This latter group recognize nursing functions, including prevention, research and education, terms that were not mentioned by incoming students. Something similar occurs with the word “hospital”, which has high frequency for incoming students and does not appear with outgoing students. This could indicate that incoming students think of the nursing profession as centered on the hospital environment, limiting workplace contexts. Similar results were obtained by Albar and Sivianes-Fernández, where fourth year nursing students differed from first year students in their perceptions of professional roles regarding research and academic development.(8) The findings suggest that incoming students have less knowledge of the tasks and functions of a nurse, even after having chosen the program, reflecting the image of nurses that society has in general.

We conclude by pointing out that, in agreement with Emeghebo, self-image and perception of nurses change throughout one’s professional program.(11) Through education, new elements are integrated into the existing structure of the representation, transforming it with growing complexity. In this study, we observe how the differences in representations of nurses held by incoming and outgoing students relate to the tasks and functions of a nurse, to the autonomous character of his or her role, to the value given to nursing as a profession and to the diversity of nursing roles that go beyond the hospital environment. We propose that future research include comparisons between incoming students’ vision and that of the non-healthcare public in general.
